# Identification of Potential Pathways of *Morella cerifera* Seedlings in Response to Alkali Stress via Transcriptomic Analysis

**DOI:** 10.3390/plants11081053

**Published:** 2022-04-12

**Authors:** Yun Jiao, Rang-Jin Xie, Hui-Min Jia

**Affiliations:** 1 Institute of Forestry, Ningbo Academy of Agricultural Science, Ningbo 315040, China; 2 Citrus Research Institute, Chinese Academy of Agricultural Sciences, Southwest University, Chongqing 400716, China; xierangjin@163.com; 3 College of Agronomy, Jiangxi Agricultural University, Nanchang 330045, China; jiahuimin1988@163.com

**Keywords:** *Morella cerifera*, alkali stress, transcriptomic analysis, gene set enrichment analysis

## Abstract

Alkali stress, a type of abiotic stress, severely inhibits plant growth. Only a few studies have investigated the mechanism underlying the transcriptional-level response of *Morella cerifera* to saline-alkali stress. Based on RNA-seq technology, gene expression differences in the fibrous roots of *M. cerifera* seedlings exposed to low- and high-concentration alkali stress (LAS and HAS, respectively) were investigated, and the corresponding 1312 and 1532 alkali stress-responsive genes were identified, respectively. According to gene set enrichment analysis, 65 gene sets were significantly enriched. Of these, 24 gene sets were shared by both treatment groups. LAS and HAS treatment groups exhibited 9 (all downregulated) and 32 (23 downregulated) unique gene sets, respectively. The differential gene sets mainly included those involved in trehalose biosynthesis and metabolism, phospholipid translocation, and lignin catabolism. Kyoto Encyclopedia of Genes and Genomes pathway analysis revealed that *M. cerifera* seedlings were specifically enriched in stilbenoid, diarylheptanoid, and gingerol biosynthesis; phenylalanine, tyrosine, and tryptophan biosynthesis; and sesquiterpenoid and triterpenoid biosynthesis. Moreover, the related genes involved in hormone signaling pathways and transcription factors were determined through a localization analysis of core abiotic stress pathways. These genes and their molecular mechanisms will be the focus of future research.

## 1. Introduction

*Morella cerifera* (L.) Small, the Wax Myrtle, an important salt and alkali-tolerant plant [1], has been gradually promoted as an excellent rootstock for the specialty fruit Chinese bayberry (*Morella rubra*) in the tidal flat area of Zhejiang Province. However, knowledge regarding the molecular regulation mechanism underlying its alkali resistance is limited [2]. The effects of alkali stress on plants are multi-faceted, affecting the entire plant life-cycle including damage to plant leaves, intracellular enzyme activities, photosynthesis, and nutrient content. This stress also affects plant organ development and interferes with plant metabolism and root absorption and the utilization of ions by roots [3]. At present, few relevant studies have investigated the effects of soil alkalinization on plant growth and development, and the mechanisms of sensing, transduction, and response to alkali stress signals remain unclear. Transcriptomic analysis is a powerful approach to understanding plant biological functions, such as growth, development, and stress response [4,5]. Identifying the alkali stress-responsive genes can help determine the genetic basis of plant alkali stress resistance through transcriptomic analysis and provide valuable information for improving crop resistance to alkali stress through genetic engineering.

Alkali stress is an extremely complex type of stress that negatively affects plants through chemical damage, osmotic stress, ionic damage, nutrient deficiency, and hypoxia. The RNA-seq analysis of rice seedlings Dongdao-4 (tolerant) and Jigeng-88 (sensitive) with different salt-alkali stress tolerances detected 3523 and 4066 genes with different expression levels, respectively. The upregulated genes in stress response were involved in jasmonic acid response, organic acid metabolism, nicotinamide biosynthesis, and iron homeostasis, while the downregulated genes were involved in photosynthesis and response to reactive oxygen species. According to the Kyoto Encyclopedia of Genes and Genomes (KEGG) pathway analysis, genes involved in diterpenoid and phenylpropane biosynthesis pathways may contribute to the stronger tolerance of Dongdao-4 to saline-alkali stress compared to Jigeng-88 [6]. In addition, carbohydrate metabolism, fatty acid accumulation, and amino acid metabolism are crucial metabolic pathways activated in canola roots exposed to alkali stress [7]. Notably, various plant hormones and transcription factors can confer tolerance to alkaline stress [8]. Among them, abscisic acid (ABA) and ethylene (ET) act as signaling molecules to activate stress-responsive genes, such as the *rab*-related (responsive to ABA) gene, RAB18 [9], and ethylene response factor (ERF)-like genes [10], thereby enhancing plant tolerance to alkaline stress. The activated transcription of stress-responsive genes enhances plant tolerance to salinity and alkali, while the overexpression of ChbZIP1, a novel bZIP transcription factor from the basic region-leucine zipper family, improves plant adaptation to alkali stress through the reactive oxygen species’ detoxification pathway [11]. The *S*-adenosylmethionine (SAM) synthase gene SISAM1 regulates the polyamine metabolism to improve the alkali tolerance of tomato [12]. Different hormone regulatory pathways and transcriptome factors interact together to respond to alkaline stress. In summary, the aforementioned research conclusions offer crucial genetic resources for the in-depth future study of alkali stress.

Currently, the soil salinization and alkalization of approximately 950 million hectares of saline land worldwide has become a major environmental threat to the entire terrestrial ecosystem, and this number continues to increase, with up to 50% of agricultural land ex-pected to be lost due to salinization by 2050 [13]. Numerous studies have investigated various aspects of the salt and alkali tolerance of plants and have reached a certain breadth and depth. However, plant alkali tolerance is a complex quantitative trait, controlled by multiple genes, and is affected by plant species, cultivar, genotype, and internal physiological and biochemical reactions. Thus, studying the alkali resistance mechanism of *M. cerifera* for the effective use of saline-alkali land is of great practical significance. In this study, transcriptomes of *M. cerifera* growing under different alkali concentrations with Na_2_CO_3_ and NaHCO_3_ (50 and 100 mmol·L^−1^) for 24 h were analyzed through high-throughput RNA-sequencing technology. The transcriptional pathways were identified through gene set enrichment analysis (GSEA), and several key alkali-responsive genes were also verified through quantitative reverse transcription-PCR (qRT-PCR).

## 2. Results

### 2.1. Differentially Expressed Genes

**Figure 1:**
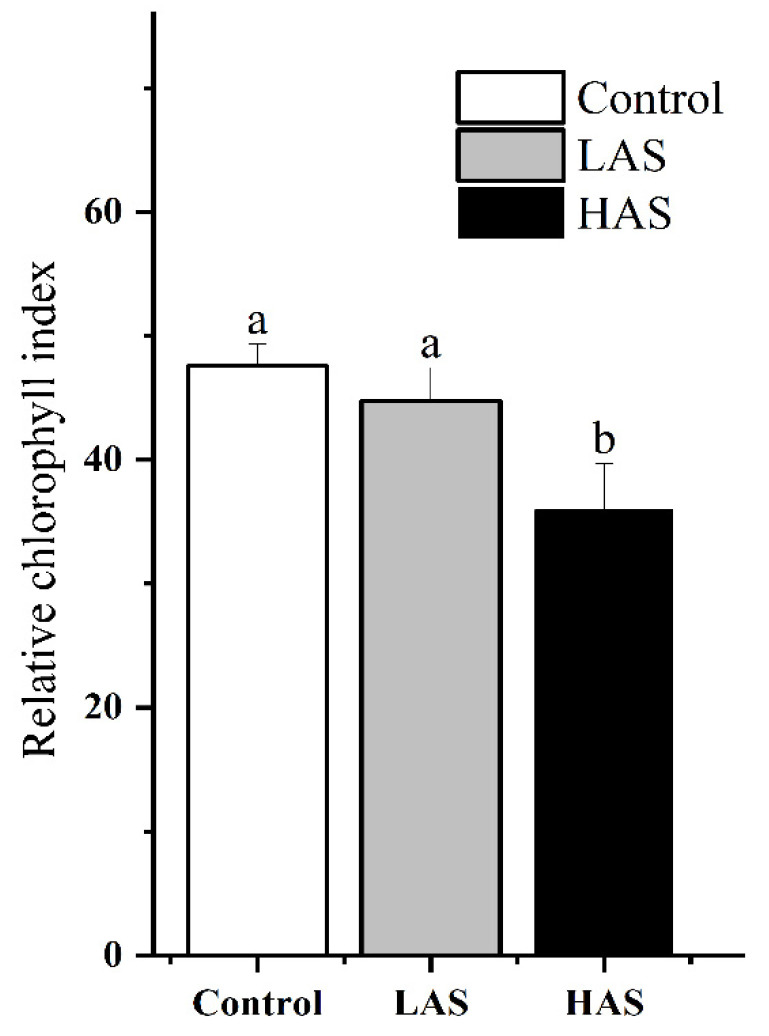
The effect of different concentration alkali stress on the leaf relative chlorophyll index of *M. cerifera*. Control, alkali stress control group; LAS, low-concentration alkali stress treatment group of *M. cerifera*; HAS, high-concentration alkali stress treatment group of *M. cerifera*. Significant differences (*p* < 0.05) were indicated by different lowercase letters according to the one-way ANOVA followed by a Duncan’s test.

**Figure 2:**
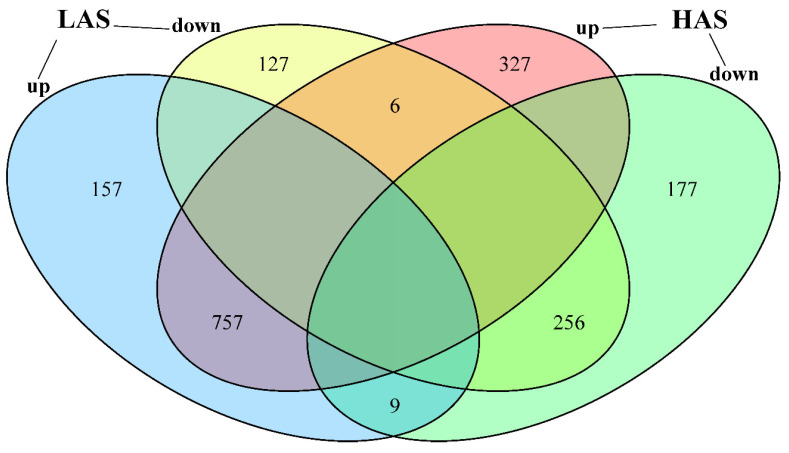
Venn diagram showing the enriched genes (upregulated and downregulated) in *M. cerifera* after low-concentration alkali stress (LAS) and high-concentration alkali stress (HAS) treatments, compared with the control with a *p* value of <0.05.

Nine sequencing libraries were constructed using RNA extracted from the fibrous roots of *M. cerifera* exposed to alkali stress, and the effects of alkali stress treatment on gene expression in the root zone of these plants were analyzed. The percentage of Q30 readings in all groups ranged from 94.04% to 96.64% (Table 1). In addition, 84–86% of the *M. cerifera* sequencing data was mapped to the Chinese bayberry reference genome. The morphological characteristics of plants exposed to LAS treatment for 24 h showed no significant change (Figure S1), whereas the HAS treatment caused the leaves to turn brown, shrink and wither at the top of the plant. Similarly, the HAS treatment resulted in a reduction in the relative chlorophyll index to the lowest levels (Figure 1), and showed significant change (*p* < 0.05) compared with the control. In addition, after 24 h of alkali stress, 1312 genes (923 upregulated and 389 downregulated) were expressed in *M. cerifera* seedlings from the LAS group. The number of differentially expressed genes (DEGs) was higher in the HAS group than in the LAS group, which was 1532 genes (1090 upregulated and 442 downregulated) (Figure 2). Meanwhile, 757 (92%) DEGs were upregulated and 256 (92%) were downregulated after both LAS and HAS treatments. Thus, these DEGs with similar expression patterns may exhibit a more consistent response regulation pattern to alkaline stress. The number of upregulated genes in response to alkali stress was higher than that of downregulated genes, which is characteristic of the root system of *M. cerifera*. Notably, 9 and 6 DGEs that were upregulated and downregulated, respectively, after LAS treatment showed the opposite expression patterns after HAS treatment. This indicates that different regulatory mechanisms may have been induced for different alkali stress concentrations, but further verification is required.

### 2.2. Gene Ontology (GO) Enrichment Analysis

**Figure 3:**
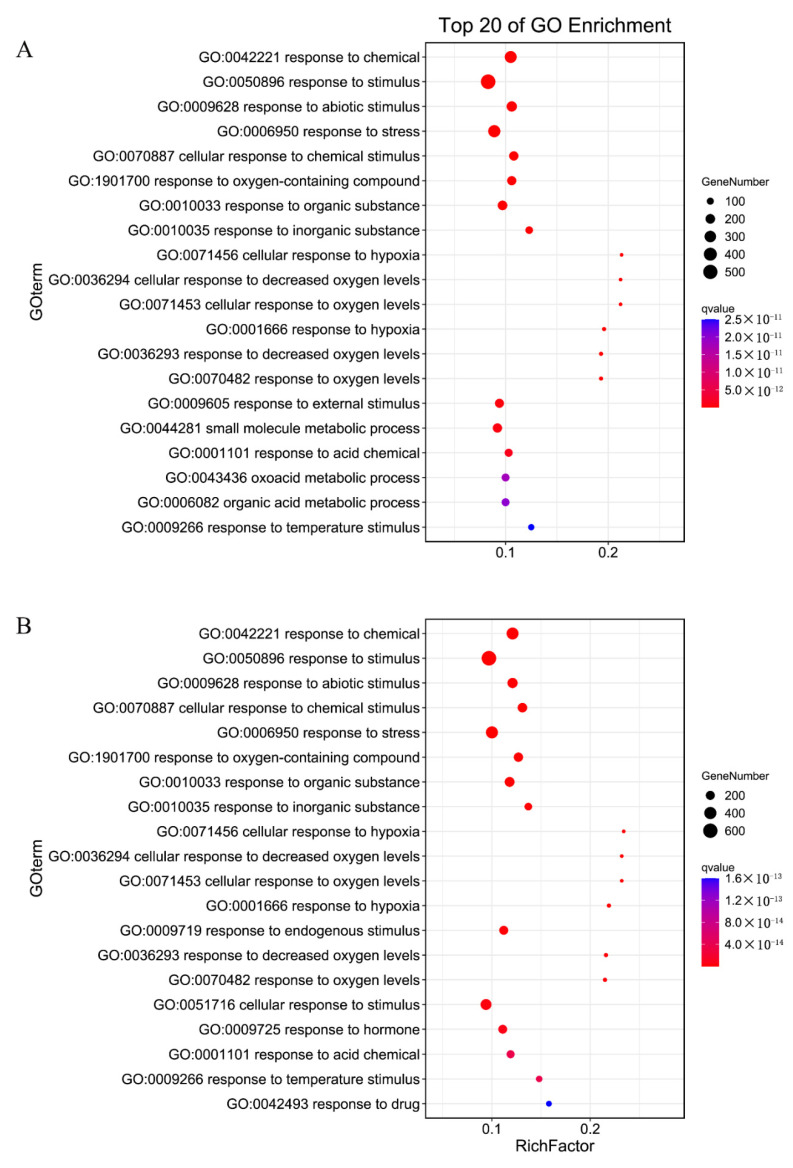
Top 20 enriched GO terms of *M. cerifera* after two treatments ((**A**), LAS; (**B**), HAS). Rich factor indicates the degree of enrichment, that is, the *p* value after multiple hypothesis testing and ranges between 0 and 1. The closer the *p* value is to zero, the more significant the enrichment.

A scatter plot of significantly enriched functions (top 20) was constructed for the GO terms identified in *M. cerifera* (Figure 3). Among them, 16 GO terms detected in the LAS and HAS groups were mainly responsible for chemical reactions (GO: 0042221), response to stimulus (GO: 0050896), response to stress (GO: 0006950), and organic matter response (GO:0010033), and these GO terms contained a large number of genes. Notedly, the hypoxia-related GO terms (GO: 0071456, GO: 0036294, GO: 0071453, GO: 0001666, GO: 0036293, and GO: 0070482) were enriched, which implied that a close relationship may exist between alkali stress and the hypoxia response. Furthermore, four GO terms were unique (GO:0044281, GO:0009605, GO:0006082, and GO:0043436) to the LAS group, and four GO terms were unique to the HAS group (GO:0051716, GO:0042493, GO:0009719, and GO:0009725), which means that the genes contained in these GO terms may respond differently to different alkali concentrations. However, differences in the response patterns of *M. cerifera* roots to alkali were generally similar between different alkali concentrations.

### 2.3. Gene Set Enrichment Analysis

**Figure 4:**
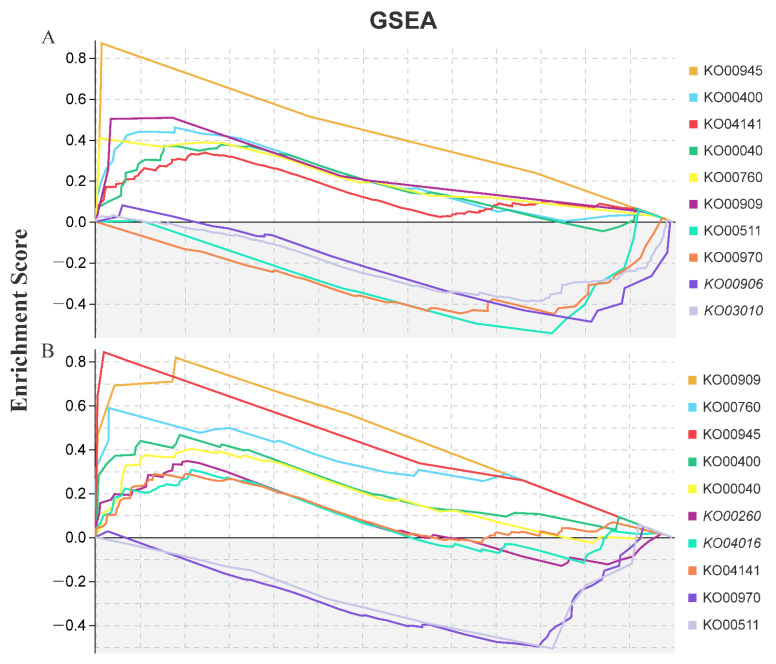
Enriched KEGG terms according to gene set enrichment analysis (GSEA) tools in *M. cerifera* after LAS and HAS treatments ((**A**), LAS; (**B**), HAS). The gene sets with a normal *p* value of <0.05 and FDR rate of <0.25 were considered significantly enriched.

Using GSEA tools, a total of 65 key gene sets were significantly enriched (Table 2). Of these, 24 gene sets were shared by LAS and HAS (3 upregulated and 19 downregulated). Moreover, with 9 unique gene sets from LAS (all downregulated) and 32 from HAS (9 upregulated and 23 downregulated), an overall trend of downregulated expression was observed. Obviously, the HAS treatment activated upregulated expression of some gene sets in response to alkali stress, including those responsible for trehalose biosynthesis and metabolism (GO:0005992, GO:0005991 and GO:0004805), phospholipid translocation (GO:0046271), and lignin decomposition metabolic processes (GO:0046274); these genes may actively repair alkaline stress-induced damage. In addition, the KEGG-GSEA pipeline used to determine the top 10 KEGG pathways for the LAS and HAS groups revealed that eight pathways, namely, stilbenoid, diarylheptanoid, and gingerol biosynthesis; phenylalanine, tyrosine, and tryptophan biosynthesis; and sesquiterpenoid and triterpenoid biosynthesis, were shared by these two treatment groups (Figure 4). Among them, most pathways showed an upregulated expression, except for aminoacyl-tRNA biosynthesis (KO00970) and glycan degradation (KO00511), which showed a downregulated expression.

### 2.4. Overview of Core Pathways and qRT-PCR Verification

**Figure 5:**
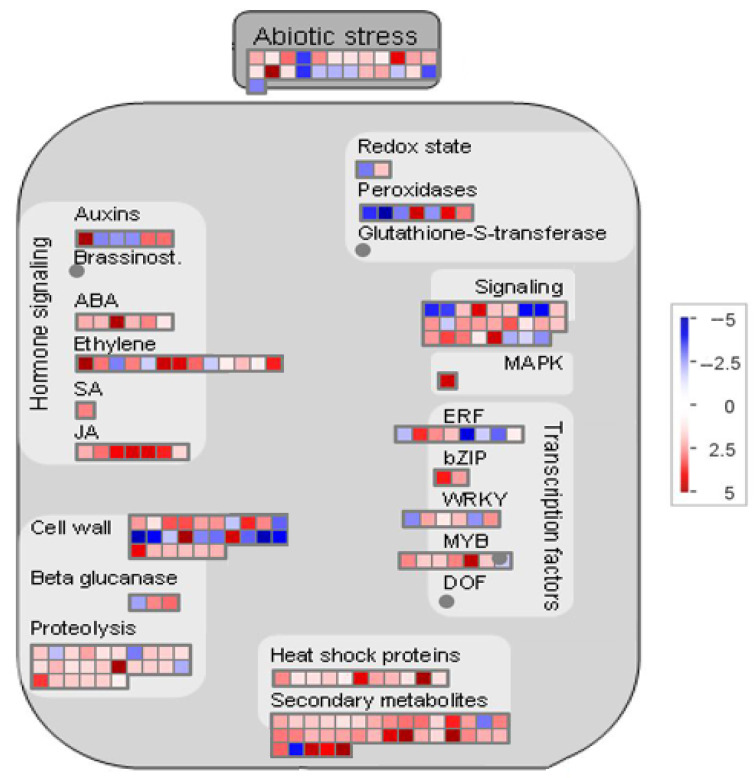
Overview of the abiotic stress pathway of genes responsive to alkali stress treatments in *M. cerifera*. Brassinosteroid (brassinost., BR), mitogen-activated protein kinase (MAPK), jasmonic acid (JA), salicylic acid (SA), ethylene response factor (ERF), basic leucine zipper (bZIP), MYB domain transcription factor (MYB), WRKY domain transcription factor (WRKY), Dof-type zinc finger domain-containing protein (DOF).

**Figure 6:**
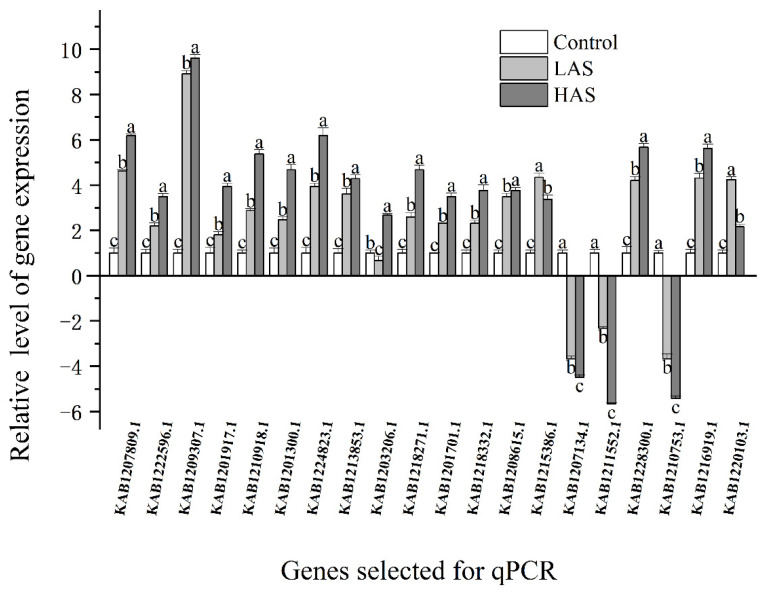
Verification of DEG expression levels through qRT-PCR. As assessed by Duncan’s multiple range test, the letters (a, b, and c) denote significant differences between treatments (*p* < 0.05).

MapMan analysis was used to identify key genes in the core pathway of *M. cerifera* response to alkali stress (Figure 5). DEGs in abiotic stress pathways were displayed by the MapMan tool, and 218 DEGs were identified after HAS treatment. Taken together, the root’s response to alkaline stress includes the expression of genes with multiple functions, including those responsible for hormone signaling, cell-wall biological processes, thermal shock proteins, and transcription factors such as ERF and WRKY domain transcription factor (WRKY). At the same time, notably, no genes were located in the three pathways, namely, brassinosteroid (brassinost.), glutathione-S-transferase and the transcription factor DOF-type zinc finger domain-containing protein, implying that these genes may not be involved in the alkali stress pathways of *M. cerifera*. Further, 20 genes were used for verification, and these genes related to hormone metabolism, cell-wall precursor synthesis, respiratory burst, peroxidases and signaling receptor kinases, which may be involved in the response of *M. cerifera* roots to alkali stress (Table S1). Finally, the qRT-PCR data were found to be consistent with the DEG data (Figure 6). On the other hand, the Pearson correlation coefficient (Cor = 0.91) and the squared multiple correlation coefficient (r^2^ = 0.88) showed a good correlation between the gene expression results obtained through qRT-PCR and those obtained through RNA-seq (Figure S2).

## 3. Discussion

Plants activate a series of complex regulatory responses to adapt to various environmental stresses. Expectedly, we also obtained GO terms in response to abiotic stimuli, chemical reactions, and stress in the GO enrichment analysis results, which were similar to the results of related studies [14,15] (Figure 3). Some GO terms related to cellular hypoxia (GO: 0036294, GO: 0071456), oxygen levels (GO: 0071453, GO:0070482), and hypoxic responses (GO: 0001666, GO: 0036293) were also found, implying that the root system is suffering from alkali stress-induced hypoxia damage. This damage is possibly due to the destruction of the cell membrane structure caused by saline-alkali toxicity and the interruption of aerobic respiration, which results in a response similar to hypoxia stress. However, no relevant research report is available to support this finding. Thus, the mechanism of alkali stress-induced cellular hypoxia requires further exploration.

We further analyzed key gene sets and regulatory pathways using GSEA-GO and KEGG (Figure 4 and Table 2). Some genes involved in secondary metabolic pathways, including phenylalanine, tyrosine, and tryptophan biosynthesis (KO00400); lignin catabolism (GO: 0046274); phenylpropane catabolism (GO: 0046271); and trehalose biosynthesis and metabolism (GO: 0005992 and GO: 0005991), were enriched, and most of them showed upregulated expression; these genes are also present in most plants. The plants are protected from stress through the mobilization of the expression of the genes that are responsible for a series of secondary metabolic pathways and antioxidant enzyme systems to eliminate accumulated reactive oxygen species [16,17,18,19]. Obviously, alkali stress also activated more gene sets involved in thylakoid membrane systems (GO:0010027, GO:0009535, GO:0055035 and GO:0042651), chloroplast structure (GO:0009706, GO:1901259 and GO: 0009941), and photosynthesis (GO:0019684, GO:0034357, and GO:0009765) were downregulated (Table 2), and may play a crucial role in the ultrastructural changes in plant leaf mesophyll cells and the photosynthetic machinery damages caused by saline-alkali stress. These gene sets are mainly involved in thylakoid degradation in mesophyll cells, chloroplast swelling, a reduction in chlorophyll and starch accumulation, and, finally, accelerated senescence of mesophyll cells [20,21,22]. In this study, the significant decrease in the leaf’s relative chlorophyll index by HAS confirmed the above speculation (Figure 1), and is similar to the related report [23], which means that they have a severe negative impact on the photosynthetic capacity of plants, and were the main reason for the shrinkage and dryness of the top leaves of the plant (Figure S1). Moreover, chorismate synthesis and metabolism (GO:0043650 and GO:0046417) was specifically enriched at the two alkali stress concentrations (Table 2). This is a major pathway for the synthesis (precursor of secondary metabolism) of tryptophan (Trp), which is widely used by higher plants in functions, including physiological processes, such as seed germination and serotonin and melatonin synthesis, and response mechanisms to biotic and abiotic stresses [24,25]. Thus, the chorismate synthesis and metabolism may be a specific response strategy used by plants to adapt to alkaline stress.

Similar to that in previous studies, KEGG-GSEA was enriched for the upregulated expression of glycine, serine, and threonine metabolic pathways (KO00260), a signal transduction event that occurs in response to environmental stress, suggesting its involvement in alkali stress [26]. They play a vital role in plant responses to abiotic stresses such as salt [27], drought [28], and cold [29]. Interestingly, this study specifically revealed the biosynthesis of stilbenoid, diarylheptanoic acid, and gingerol under alkali stress (KO00945); this pathway is vital for cotton insect resistance [30]. Thus, this pathway may be induced by biotic and abiotic stresses at the same time. In addition to the aforementioned research results, this study revealed that hemiterpenoid and triterpenoid biosynthesis pathways (KO00909) were upregulated under alkali stress. Studies have shown that triterpenoids are closely related to various plant processes, including respiration, photosynthesis, and responses to environmental stimuli. Squalene monooxidase is induced in sugar beet leaves at low concentrations of neutral and alkaline salt [31]. It is involved in the conversion of squalene into a major compound in the early stage of plant triterpenoid biosynthesis-2,3-oxyquinene, which may be involved in growth promotion. However, its role in alkali stress needs to be explored.

Further, abiotic stress core pathway analysis identified genes related to hormone signaling, including ethylene, auxin, abscisic acid, jasmonic acid, and salicylic acid. These genes were induced and are mainly upregulated under alkali stress [32,33]. As indicated in the related literature, they may balance the osmotic potential and reduce plasma membrane permeation and peroxidation by regulating the accumulation of cellular ions such as Na^+^, K^+^, and Ca^2+^ and compatible metabolites such as proline and soluble sugars, thereby increasing alkali resistance in plants [34]. Therefore, exogenous hormones such as salicylic acid can be used to effectively enhance the tolerance of plants in saline-alkali soil. In addition, transcription factors are key regulators of gene expression, with crucial functions in plant responses to abiotic stresses [35]. Unsurprisingly, the abiotic stress core pathway also localized the differential expression of many transcription factors under alkaline stress, especially transcription factor families such as WRKY, bZIP, ERF, and MYB. The results of transcriptome studies in yellow horn (*Xanthoceras sorbifolia*) [36], *Sophora alopecuroides* [37], and switchgrass (*Panicum virgatum* L.) [38] revealed that the expression levels of transcription factors such as WRKY, ERF, DEREB, MYB, and NAC changed significantly under alkaline stress. These transcription factors were highly enriched among DEGs under alkaline stress, suggesting that they are jointly involved in the regulation of myricetine stress resistance. In conclusion, the new biological pathways and candidate genes identified in this study on the alkali tolerance of *M. cerifera* will more comprehensively explain the molecular mechanism underlying alkali tolerance in plants.

## 4. Materials and Methods

### 4.1. Plant Treatment and Measurements of Physiological Characteristics

In this study, seeds (>200) of disease-free *M. cerifera* were collected at the experimental base of Ningbo Academy of Agricultural Sciences in January 2020 and were directly sown in soil containing perlite and peat (1:1, *v*/*v*). Evenly sized seedlings were collected and transferred to plastic hydroponic containers (31 × 29 × 18 cm, 18 plants equally spaced in each pot) until May 2021. In the hydroponic container, the nutrient solution (half-strength Hoagland’s solution) [18] was injected to 0.5 cm above the junction of the stem and root and fixed with a sponge. The root zone was aerated using an air pump with constant bubbling, and the solution was changed every 5 days. The seedlings were grown in a glass greenhouse under natural light, and the indoor air temperature was maintained at 25 °C ± 2 °C with a relative humidity (RH) of 70% ± 5% by using an air-conditioning system. In the treatment groups, the plants were exposed to alkali stress after 20 days of stable culture. Specifically, plant rootstocks were divided into three groups: control (“Control”, 1/2 Hoagland nutrient solution without alkali), low-concentration alkali treatment (“LAS”, 1/2 Hoagland nutrient solution containing a 1:9 mix of Na_2_CO_3_ and NaHCO_3_; final Na^+^ concentration: 50 mM; pH 8.5), and high-concentration alkali treatment (“HAS”, 1/2 Hoagland nutrient solution containing a 1:9 mix of Na_2_CO_3_ and NaHCO_3_; final Na^+^ concentration: 100 mM; pH 8.9). Each group was replicated three times, and every six strains was a replicate. After the plants were exposed to stress through treatment with alkaline solution for 24 h, three groups of treated fibrous root samples were taken separately, carefully washed with double-distilled water, and then immediately frozen in liquid nitrogen for further analysis. In addition, a Dualex 4 (FORCE-A, Orsay, France) was used to measure the relative chlorophyll index of *M. cerifera* leaves using the nitrogen balance index (NBI), and 20 leaves were measured for each treatment [39].

### 4.2. RNA-Sequencing and Assembly

Total RNA was extracted from the fibrous roots using the Plant Total RNA Isolation Kit (Aidlab Biotechnologies, Beijing, China), and RNA quality was then detected using the NanoDrop 2000 spectrophotometer (Thermo Fisher Scientific, Waltham, MA, USA). The RNA was further purified with the RNase-Free DNase Set (Qiagen, Hilden, Germany). Next, RNA-seq libraries were constructed using the TruSeq RNA Sample Prep Kit (Illumina, San Diego, CA, USA) and employed for high-throughput sequencing using the Illumina HiSeq 4000 platform (Illumina, San Diego, CA, USA) to generate 150-bp paired-end reads. Raw sequencing data files (56.3 GB) were uploaded to the NCBI Sequence Read Archive database (project number: PRJNA768144). To remove low-quality bases, raw reads were filtered using Trimmomatic [40]. All clean reads were mapped to the Chinese bayberry genome (https://www.ncbi.nlm.nih.gov/assembly/GCA_003952965.2, accessed on 25 November 2021), which was used as the reference genome. Using the transcriptome sequencing data, the expression level of the corresponding gene was estimated on the basis of the number of clean reads. Transcript abundance was normalized by fragments per kilobase per million parameters [41].

### 4.3. DEGs and GSEA Analysis

The software edgeR [42] was used to screen for DEGs between the two groups (control and alkali treatment), and the false discovery rate (FDR) *p*-value < 0.05 criterion was used to identify DEGs. Next, the GSEA analysis tool was used to identify DEG functions, and the GO annotation file corresponding to *Arabidopsis thaliana* (https://www.arabidopsis.org/download/index.jsp, accessed on 7 December 2021). CLC Genomics Workbench software version 12.0 was used to perform native BLAST functions to create gene set files [43]. Then, KEGG pathways were identified and enriched using the OmicShare GSEA tool (https://www.omicsshare.com/tools/, accessed on 22 December 2021) with a *p* value of <0.01 and FDR of <0.20. Finally, DEG pathways were mapped and analyzed using MapMan software version 3.6.0 (http://mapman.gabipd.org/web/guest, accessed on 11 December 2021) [44,45].

### 4.4. qRT-PCR Analysis

To confirm RNA-seq reliability, 20 key DEGs were selected, and real-time quantitative PCR was used to verify their expression under the two treatments. Primers with an amplification length of approximately 120 bp were designed using Primer Premier 5.0 software [46] (Table S1), and SYBR Premix Ex TaqTM (TaKaRa Bio, Shiga, Japan) was used for real-time quantitative PCR analysis. To ensure the accuracy of results, three biological and three technical replicates were used for each gene for validation, and the average Ct value was used to analyze changes in expression. Data were normalized with the actin gene [47]. qRT-PCR was run using the ABI Q6 Flex real-time PCR system (Applied Biosystems, Foster City, CA, USA), and the cycle parameters and reaction system composition for this run were as per the manufacturer’s instructions. qRT-PCR result data were exported to Microsoft Excel 365 (Microsoft Corporation, Redmond, WA, USA) and processed using Data Processing System software version 14.10 (Zhejiang University, Hangzhou, China) [48] for Duncan’s test (*p* < 0.05) variance analysis, and investigate the correlation coefficient with the date of the RNA-seq. Finally, the graphics were drawn using OriginPro version 2021 (Northampton, MA, USA).

## 5. Conclusions

In conclusion, 1312 and 1532 alkali stress-responsive genes were identified; changes in the expression of these genes were detected in the roots of M. cerifera exposed to stress with two alkali concentrations (LAS and HAS), respectively. These genes were responsible for cellular hypoxia; stilbenoid, diarylheptanoid, and gingerol biosynthesis; sesquiterpenoid and triterpenoid biosynthesis; hormone signaling pathways; and transcription factors. The effects of these genes on myricetine stress resistance and their molecular mechanisms will be the focus of future research. This study enriches our knowledge about plant response to alkaline stress at the molecular level and provides new insights into plant resistance to this stress.

## Figures and Tables

**Table 1:** Summary of read counts and total bases for various alkali stress groups in *M. cerifera*. Control, alkali stress control group; LAS, low-concentration alkali stress treatment group of *M. cerifera*; HAS, high-concentration alkali stress treatment group of *M. cerifera*.

Treatment	Total Read Count	Q20 (%)	Q30 (%)	Total Mapped
Control	53,821,293	98.75%	94.04%	86%
LAS	56,937,296	98.96%	94.79%	86%
HAS	54,063,143	98.25%	96.64%	84%

**Table 2:** Terms of enriched gene sets associated with alkali stress in *M. cerifera* determined using GSEA. This table shows NES obtained from GSEA for those gene sets with significant enrichment (FDR q-value < 0.20 and NOM *p*-value <  0.01). The superscripts “a” and “b” represent GO terms significantly enriched in LAS and HAS groups, respectively, while superscript “c” represents GO terms shared by the two treatment groups.

Gene Set ID	GO Term	Gene Set Size	NES	
			LAS	HAS
GO:0009943 ^a^	Adaxial/abaxial axis specification	18	−1.77	-
GO:0051187 ^a^	Cofactor catabolism	73	−1.75	-
GO:0010027 ^a^	Thylakoid membrane organization	40	−1.76	-
GO:0008252 ^a^	Nucleotidase activity	11	−1.77	-
GO:0017001 ^a^	Antibiotic catabolism	63	−1.74	-
GO:0010497 ^a^	Plasmodesmata-mediated intercellular transport	9	−1.71	-
GO:0015772 ^a^	Oligosaccharide transport	7	−1.81	-
GO:0009668 ^a^	Plastid membrane organization	41	−1.72	-
GO:0015770 ^a^	Sucrose transport	6	−1.82	-
GO:0046417 ^c^	Chorismate metabolism	10	2.34	2.16
GO:0005977 ^c^	Glycogen metabolism	17	−1.80	−1.86
GO:0006112 ^c^	Energy reserve metabolism	17	−1.81	−1.88
GO:0032544 ^c^	Plastid translation	15	−1.75	−1.82
GO:1905393 ^c^	Plant organ formation	82	−1.73	−1.80
GO:0016810 ^c^	Hydrolase activity, acting on carbon–nitrogen (but not peptide) bonds	79	−1.91	−1.79
GO:0019684 ^c^	Photosynthesis, light reaction	83	−1.74	−1.79
GO:0003002 ^c^	Regionalization	112	−1.73	−1.74
GO:0000313 ^c^	Organellar ribosome	34	−1.73	−1.74
GO:0072598 ^c^	Protein localization to chloroplast	34	−1.73	−1.73
GO:0009706 ^c^	Chloroplast inner membrane	71	−1.75	−1.73
GO:0016811 ^c^	hydrolase activity, acting on carbon–nitrogen (but not peptide) bonds, in linear amides	41	−1.74	−1.72
GO:0044436 ^c^	Thylakoid part	314	−1.76	−1.69
GO:0009528 ^c^	Plastid inner membrane	73	−1.78	−1.72
GO:0009535 ^c^	Chloroplast thylakoid membrane	284	−1.79	−1.70
GO:0055035 ^c^	Plastid thylakoid membrane	285	−1.79	−1.70
GO:0034357 ^c^	Photosynthetic membrane	300	−1.77	−1.68
GO:0009534 ^c^	Chloroplast thylakoid	358	−1.73	−1.68
GO:0048449 ^c^	Floral organ formation	15	−1.71	−1.69
GO:0009579 ^c^	Thylakoid	410	−1.80	−1.68
GO:0043650 ^c^	Dicarboxylic acid biosynthesis	21	2.27	2.05
GO:0042651 ^c^	Thylakoid membrane	300	−1.77	−1.69
GO:0031976 ^c^	Plastid thylakoid	359	−1.73	−1.68
GO:0009423 ^c^	Chorismate biosynthesis	6	2.10	1.95
GO:0005992 ^b^	Trehalose biosynthesis	10	-	2.32
GO:0005991 ^b^	Trehalose metabolism	12	-	2.21
GO:0019843 ^b^	rRNA binding	86	-	−1.90
GO:0000786 ^b^	Nucleosome	23	-	−1.87
GO:0006333 ^b^	Chromatin assembly or disassembly	31	-	−1.83
GO:0045332 ^b^	Phospholipid translocation	6	-	2.04
GO:0046271 ^b^	Phenylpropanoid catabolism	10	-	2.05
GO:1901259 ^b^	Chloroplast rRNA processing	13	-	−1.77
GO:1905268 ^b^	Negative regulation of chromatin organization	18	-	−1.77
GO:0009522 ^b^	Photosystem I	24	-	−1.78
GO:0046274 ^b^	Lignin catabolic process	10	-	2.05
GO:0045815 ^b^	Positive regulation of gene expression, epigenetic	10	-	−1.77
GO:0009765 ^b^	Photosynthesis, light harvesting	22	-	−1.77
GO:0007389 ^b^	Pattern specification process	138	-	−1.78
GO:0046351 ^b^	Disaccharide biosynthesis	20	-	2.01
GO:0031204 ^b^	Posttranslational protein targeting to membrane, translocation	7	-	1.99
GO:0046658 ^b^	Anchored component of the plasma membrane	95	-	−1.75
GO:0042447 ^b^	Hormone catabolism	12	-	2.05
GO:0016584 ^b^	Nucleosome positioning	7	-	−1.74
GO:0031491 ^b^	Nucleosome binding	17	-	−1.75
GO:0031492 ^b^	Nucleosomal DNA binding	5	-	−1.70
GO:0046113 ^b^	Nucleobase catabolism	8	-	−1.71
GO:0072596 ^b^	Establishment of protein localization to chloroplast	32	-	−1.71
GO:0009941 ^b^	Chloroplast envelope	500	-	−1.71
GO:0004805 ^b^	Trehalose-phosphatase activity	6	-	1.97
GO:0010206 ^b^	Photosystem II repair	8	-	−1.69
GO:0031936 ^b^	Negative regulation of chromatin silencing	9	-	−1.69
GO:0009798 ^b^	Axis specification	32	-	−1.72
GO:0051053 ^b^	Negative regulation of DNA metabolism	32	-	−1.68
GO:0030145 ^b^	Manganese ion binding	28	-	−1.71
GO:0045036 ^b^	Protein targeting the chloroplast	32	-	−1.71
GO:0000018 ^b^	Regulation of DNA recombination	20	-	−1.68

## Data Availability

All data have been included in the main text.

## Supplementary Materials

The following supporting information can be downloaded at: https://www.mdpi.com/article/10.3390/plants11081053/s1, Figure S1: Morphological changes in leaves of *M. cerifera* after 24-h alkali stress treatment (control, LAS, and HAS). Figure S2: The correlation between the gene expression results obtained through qRT-PCR (20 key DEGs, shown in Figure 6) and those obtained through RNA-seq, which can be calculated as the Pearson correlation coefficient (Cor) and the squared multiple correlation coefficient (r^2^). Table S1: The primers for qRT-PCR of the genes. The key genes involved in the abiotic stress pathway of *M. cerifera* under alkali stress.
