# Supplementary material for: Identification of Potential Pathways of Morella cerifera Seedlings in Response to Alkali Stress via Transcriptomic Analysis
Source: Plants (Basel). 2022 Apr 12;11(8):1053. doi: 10.3390/plants11081053 (PMC9026155; doi:10.3390/plants11081053)
Supplement: Supplementary file 1 [file plants-11-01053-s001.zip › plants-1623658-supplementary.pdf]

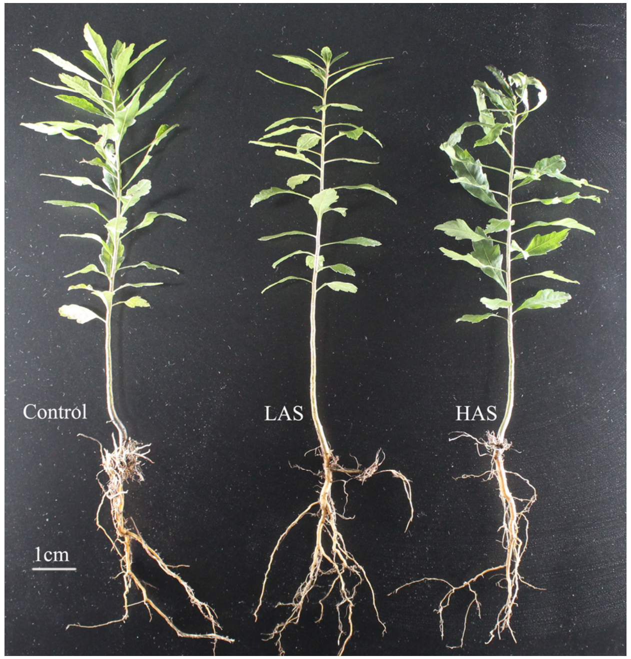

Figure S1. Morphological changes in leaves of *M. cerifera* after 24-h alkali stress treatment (control, LAS, and HAS).

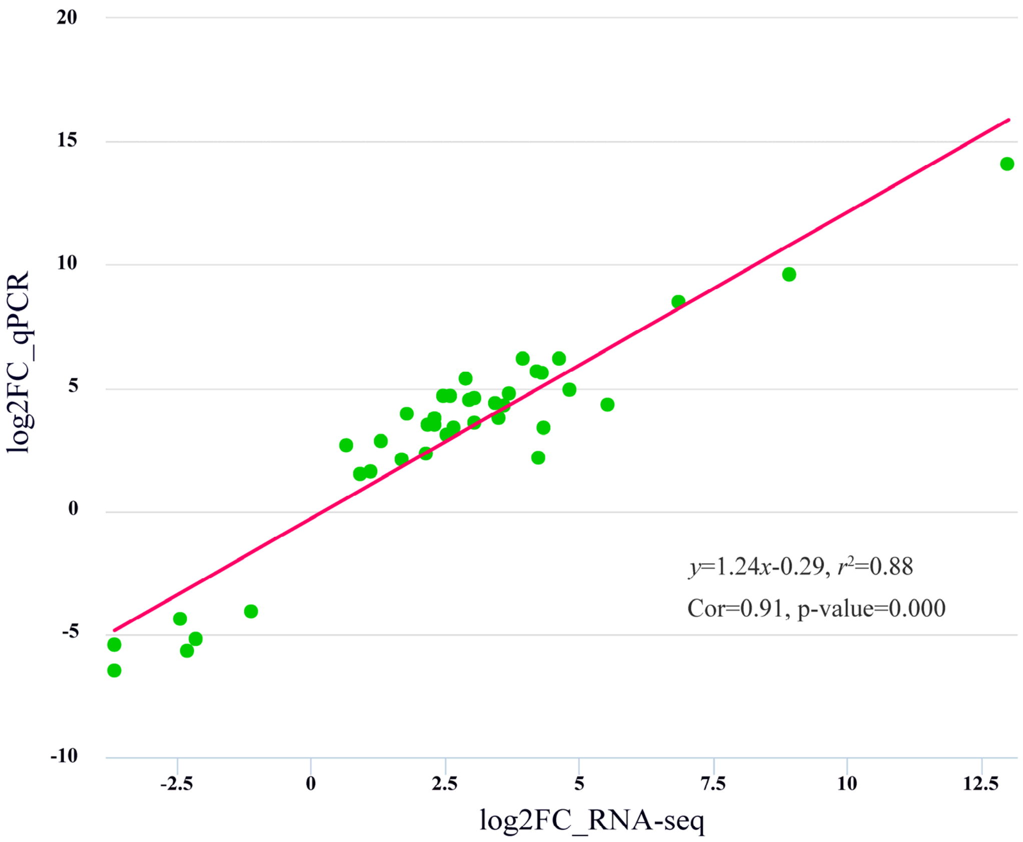

Figure S2. The correlation between the gene expression results obtained through qRT-PCR (20 key DEGs, shown in Figure 6) and those obtained through RNA-seq, which can be calculated as the Pearson correlation coefficient (Cor) and the squared multiple correlation coefficient (r^2).

Table S1. The primers for reverse transcription-quantitative PCR (RT-qPCR) of the genes. The key genes involved in the abiotic stress pathway of *Morella cerifera* with alkali stress.

Gene code	Primer Sequence (5'→3')	Target gene description	Locus ID*
KAB1207809.1-F	TTGGACGGAGCTGATTGGAT	Hormone metabolism.auxin.synthesis-degradation. Encodes a UDP-glucose:thiohydroximate	AT1G24100.1
KAB1207809.1-R	GTAAGCCGATGGCACCATTG	S-glucosyltransferase	
KAB1222596.1-F	CGAAGACTGCAGACCCAAAAG	Hormone metabolism.abscisic acid.synthesis-degradation.synthesis.9-cis-epoxycarotenoid dioxygenase	AT1G30100.1
KAB1222596.1-R	GGTAGACGCCGTTGATGCAT		
KAB1209307.1-F	TCGGGAAGCAACGGATGA	Hormone metabolism.ethylene.synthesis-degradation.1-aminocyclopropane-1-carboxylate oxidase	AT2G19590.1
KAB1209307.1-R	CTCCCCGAGAATGCTTCCA		
KAB1201917.1-F	GGAGGACGAATGGTGCTGAT	Hormone metabolism.salicylic acid.synthesis-degradation	AT4G36470.1
KAB1201917.1-R	TTCTCCCTTCGAAACCAATTTG		
KAB1210918.1-F	GCTTGCCGCTAGGAATGCT	Hormone metabolism.jasmonate.synthesis-degradation.12-Oxo-PDA-reductase	AT1G76690.1
KAB1210918.1-R	CCGTATTGGTCTGTTCTATCATTCA		
KAB1201300.1-F	ATGGCAGTCATTGCACTCAAGT	Cell wall.precursor synthesis.UDP-Glc dehydrogenase (UGD)	AT3G29360.1
KAB1201300.1-R	TTCTTCAAGACCCGGCTCAT		
KAB1224823.1-F	AAACAGGGCCATGTCCAGAA	Cell wall.degradation.pectate lyases and polygalacturonases	AT3G07970.1
KAB1224823.1-R	GAATGCTCTCGCTACAATCGAA		
KAB1213853.1-F	TTTGGAACAAGCAACAGATCGT	Misc.beta 1,3 glucan hydrolases.glucan endo-1,3-beta-glucosidase	AT2G27500.1
KAB1213853.1-R	GTTGCCGAGCATTCCATAGC		
KAB1203206.1-F	TCTTCGGAGATGGTCCTTACGT	Protein.degradation.aspartate protease	AT1G03220.1
KAB1203206.1-R	AGGGCTCGCCTGCTATGTAG		
KAB1218271.1-F	CCACCACCCTGCGAAAAG	Protein.degradation.ubiquitin.E3.RING	AT1G53820.1
KAB1218271.1-R	GCTCAAGCTCAGAGAGGCAAA		
KAB1201701.1-F	ACGAGCCCATGAAACCTTACTTT	Stress.biotic.respiratory burst. NADPH/respiratory burst oxidase protein D (RbohD)	AT5G47910.1
KAB1201701.1-R	GCGTAAATGCTATGGCCATCA		

KAB1218332.1-F	GCTCCTCGCATGGACACAGT	Stress.biotic.signalling. JAZ6 transcript levels rise in response to a jasmonate stimulus and a GFP	AT1G72450.1
KAB1218332.1-R	GGCAATAGCTTGTGGCTTTTG		
KAB1208615.1-F	GCCCTTATGGCGAGAACCTT	Stress.biotic.PR-proteins. pathogenesis related protein	AT2G14580.1
KAB1208615.1-R	TGCCGCCAACACATGAATTA		
KAB1215386.1-F	TGGGTTCTACTCGGCCTACCT	Stress.abiotic.heat. Heat shock protein 83	AT5G52640.1
KAB1215386.1-R	ACGTCCCTGGTGACAGTGAAA		
KAB1207134.1-F	TCTTGACGACACCGACGATTT	Misc.peroxidases. Peroxidase superfamily protein	AT1G49570.1
KAB1207134.1-R	GTGGCAGGACAAACTCTTTCG		
KAB1211552.1-F	TACTGGCCTAGACGATGAGCAA	Signalling.receptor kinases.Catharanthus roseus-like RLK1. Encodes a synergid-expressed	AT3G51550.1
KAB1211552.1-R	AAGCTCTCCGGCGCAATTA		
KAB1228300.1-F	TTGACCCAGTGACGGTCTTGTA	Signalling.MAP kinases. member of MEKK subfamily	AT2G32510.1
KAB1228300.1-R	CCTTTGGGCTCCTCCTTAGG		
KAB1210753.1-F	AAGGCCCGTACGTTCTCATG	RNA.regulation of transcription.AP2/EREBP, APETALA2/Ethylene-responsive element binding protein	AT5G52020.1
KAB1210753.1-R	TTGCGTGGTTCTCGTATTTCG	family	
KAB1216919.1-F	ACGAATGGATTCACTGTTCGATTA	RNA.regulation of transcription.MYB domain transcription factor family	AT1G79180.1
KAB1216919.1-R	TCAACATCAGCCTCCAATGG		
KAB1215386.1-F	TTGGGTTCTACTCGGCCTACCT	Stress.abiotic.heat, Heat shock protein 83	AT5G52640.1
KAB1215386.1-R	CGTCCCTGGTGACAGTGAAA		
Actin-F	AATGGAACTGGAATGGTCAAGGC		
Actin-R	TGCCAGATCTTCTCCATGTCATCCCA		

Note: Gene code and locus ID were derived from *Morella rubra* 'Y2012-145' (https://www.ncbi.nlm.nih.gov/assembly/GCA_003952965.2) and *Arabidopsis thaliana* (TAIR10) (https://www.arabidopsis.org/index.jsp), respectively.
